# *Candida albicans* Interactions with Bacteria in the Context of Human Health and Disease

**DOI:** 10.1371/journal.ppat.1000886

**Published:** 2010-04-29

**Authors:** Diana K. Morales, Deborah A. Hogan

**Affiliations:** Department of Microbiology and Immunology, Dartmouth Medical School, Hanover, New Hampshire, United States of America; University of California San Francisco, United States of America

Humans are colonized by diverse populations of bacteria and fungi when in a healthy state and in the settings of disease, and the interactions between these microbial populations can be beneficial or detrimental to the host [1]. Among these microbial populations, *Candida albicans* is the fungus most commonly detected in association with humans [2], and numerous studies have described *C. albicans* interactions with its bacterial neighbors [1]. Here, with a focus on *C. albicans*, we provide examples of how bacterial-fungal interactions can influence human health. In addition, we highlight studies that give insight into the molecular mechanisms that govern the physical associations, interspecies communication, and changes in microbial behavior and survival that occur when bacteria and fungi occupy the same sites.

## Bacterial−*C. albicans* Interactions Can Promote or Prevent Disease

**Figure 1:**
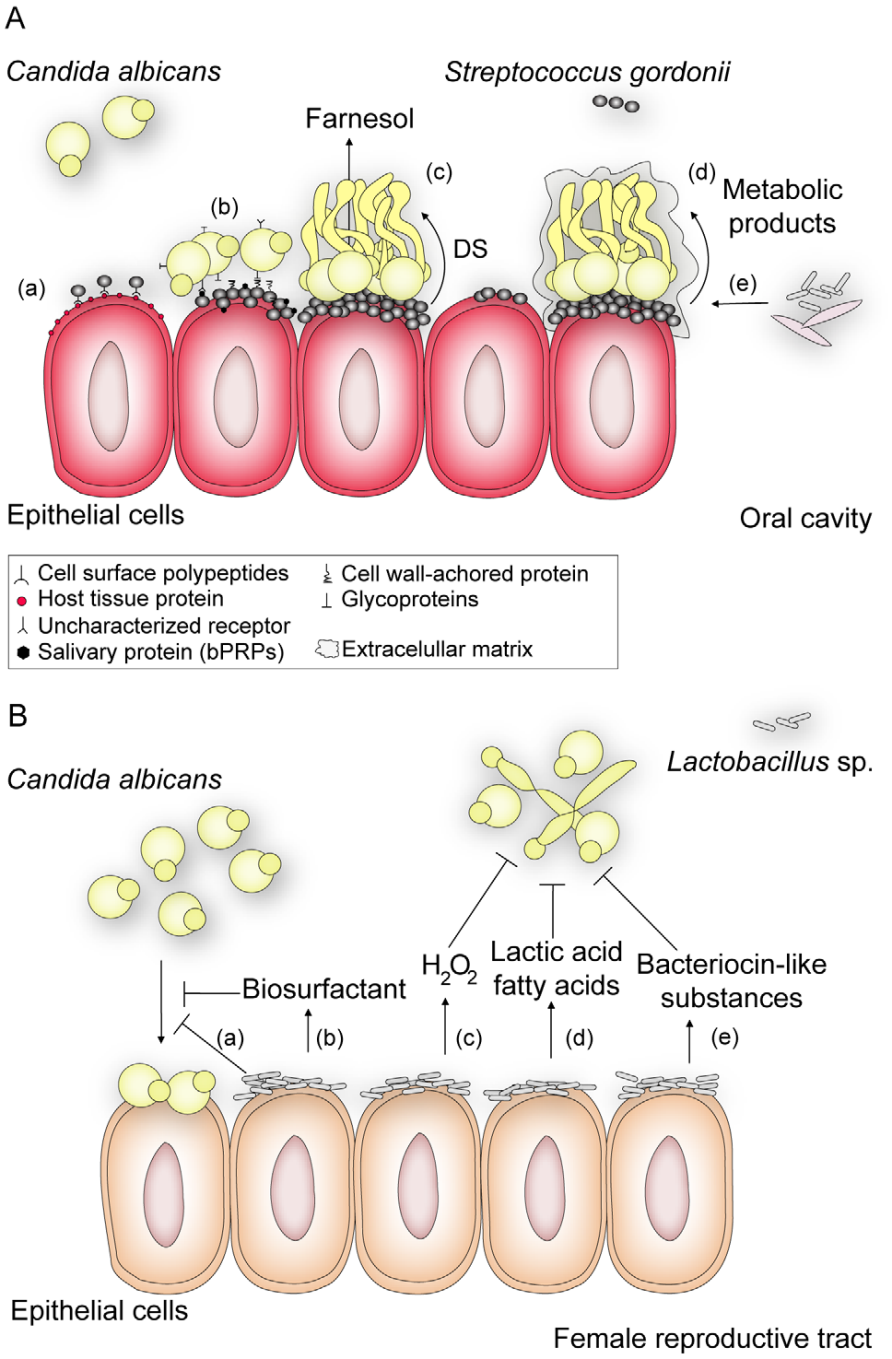
An overview of how interactions between *C. albicans* and Gram-positive members of the human flora may influence disease. (A) *S. gordonii*, a normal colonizer of the oral cavity, enhances *C. albicans* survival and persistence, thus contributing to the development of *C. albicans* infections. In this environment, *S. gordonii* adhere to cells by expressing a complex repertoire of cell surface polypeptides (AgI/II) (a) that recognize a range of host tissue proteins and cellular receptors [7]. After bacterial attachment, *C. albicans* can selectively attach to surface-bound bacteria by means of protein-protein interactions [10], [12] or by direct recognition of salivary proteins (basic proline-rich proteins, bPRPs) previously adsorbed by *S. gordonii* cells [11] (b). Coaggregation of the bacterium and *C. albicans* contributes to biofilm formation and results in closer proximity for cell-cell communication. Through a diffusible signal (DS) molecule (c), *S. gordonii* suppresses farnesol-mediated inhibition of hypha formation, thereby enhancing the potential for *C. albicans* to form biofilms and thus its ability to invade tissue [7]. In addition, *Streptococcus* promotes (d) fungal growth by secreting metabolic products that can be used as a carbon source by *C. albicans* [1]. Likewise, *C. albicans* enhances the survival and colonization of *S. gordonii* by reducing the oxygen tension to levels preferred by streptococci and by providing bacterial growth stimulatory factors as a result of nutrient metabolism [1], [11]. These favorable conditions promote the formation of mature fungal-bacterial biofilms surrounded by an extracellular matrix, to which (e) other bacterial or fungal species can bind. These interactions may make oral infections more persistent and harder to treat. (B) *Lactobacillus* sp., which normally inhabits the female reproductive tract, defends the host against colonization of pathogens such as *C. albicans*. Evidence suggests that the bacterium reduces the adhesion of *C. albicans* to epithelial cells either by (a) outcompeting fungal cells for adhesion sites, such as cellular receptors to which *Lactobacillus* has higher affinity, or (b) by secreting biosurfactants such as surlactin that physically decrease fungal binding. Most *Lactobacillus* strains release (c) hydrogen peroxide (H_2_O_2_) and (d) lactic acid or other fatty acids that inhibit *C. albicans* proliferation and invasive hypha formation. Bacteriocin-like substances (e) produced by *Lactobacillus* suppress the fungal growth to directly decrease its load [8].

Bacterial and fungal co-infections have been implicated in enhanced host colonization and virulence. For instance, *C. albicans* and *Escherichia coli* exhibit a cooperative interaction wherein *E. coli* enhances adhesion of *C. albicans* to bladder mucosa and increases the likelihood of fungal urinary tract infections [3]. Likewise, the risk of ventilator-associated pneumonia due to infection by *Pseudomonas aeruginosa* is markedly greater in patients colonized by *C. albicans* [4], and accordingly, antifungal treatments can reduce the likelihood of developing this systemic disease [5]. Moreover, denture stomatitis, an inflammation of the oral mucosa in denture wearers, is influenced by the presence of *C. albicans* and other oral microorganisms [6]. In fact, several studies demonstrate an association between *C. albicans* and oral bacteria such as *Streptococcus* (Figure 1A), *Actinomyces*, and *Fusobacterium* species [1], [7], and these physical interactions likely contribute to denture colonization and oral candidiasis.

In contrast, lactic acid bacteria (Figure 1B), which normally inhabit the intestinal and female reproductive tracts, compete with *C. albicans* for adhesion sites and secrete substances that inhibit fungal attachment to control *C. albicans* invasion and disease [8]. Interestingly, imbalance in the normal bacterial flora caused by treatment with broad-spectrum antibiotics is a predisposing factor associated with *C. albicans* colonization of immunocompromised patients, probably due to decreased numbers of bacterial competitors [9]. Thus, antibiotic therapies that specifically target pathogens, in contrast to broad spectrum antibiotics, may help prevent secondary problems that arise upon perturbation of beneficial bacterial-fungal interactions.

## Bacteria and Fungi Promote Coaggregation and Formation of Mixed-Species Biofilms

Both singly and together, bacteria and fungi form highly structured, often surface-associated, communities termed biofilms. A significant proportion of human microbial infections are biofilm-associated, wherein the formation of mixed-species biofilms could create a protected environment that allows for survival to external assaults and facilitates different bacterial-fungal interactions [1], [2]. The known relationships between *C. albicans* and oral streptococci illustrate the various ways by which bacteria and fungi can attach to one another or coaggregate using specific cell surface factors, leading to mixed-species biofilms [2], [7]. These adhesive interactions between *C. albicans* and other indigenous oral microbes can be mediated by protein-protein and lectin-carbohydrate interactions, and hydrophobic and electrostatic interactions may contribute as well. *C. albicans* molecules such as agglutinin-like sequences (Als) and specific cell surface glycoproteins have been identified as being important for coadhesion to mixed microbial communities in biofilms [1], [10]. O'Sullivan and colleagues [11] demonstrated that the oral bacterium *Streptococcus gordonii* adsorbs salivary proline-rich proteins that are recognized by *C. albicans* and act as receptors for the fungus (Figure 1A). Importantly, *S. gordonii* cell surface polypeptides also contribute to coadherance with the fungi [12]. Underscoring the complexity of these interactions, adherence and coaggregation of *C. albicans* with oral bacteria is species specific, and is mediated by bacterial receptors that might be expressed only under particular environments.

## Interdomain Signaling via Quorum-Sensing Molecules and Other Microbial Products Modulates Fungal and Bacterial Behavior

Single species bacterial and fungal populations modulate their collective behavior using extracellular signals known as quorum-sensing molecules [13], [14]. Since this regulation generally occurs in response to cell density, processes like co-aggregation and biofilm formation promote the synthesis and secretion of quorum-sensing molecules, increasing the likelihood that neighboring cells will experience the signals at levels sufficient to induce a response. Thus, the study of bacterial-fungal interactions in association with mixed-species biofilms reveals that cross-kingdom communication between bacteria and fungi is a common process, and that quorum-sensing signals, known for their roles in intraspecies communication, can also mediate crosstalk between bacteria and fungi. *C. albicans*, as an illustration, induces its switch from hyphal growth to yeast growth using a secreted quorum-sensing molecule called farnesol [14], which inhibits the Ras1-controlled pathway involved in hyphal growth [15]. Strikingly, this small molecule can also modulate bacterial behavior and virulence by altering the production of toxic phenazines, such as pyocyanin in *P. aeruginosa* [13]. Moreover, farnesol can also induce the generation of reactive oxygen species in a number of microorganisms, likely through effects on electron transport chain components [16], and this process may play an important role in competition with bacteria.

Notably, a number of Gram-negative bacteria secrete molecules with farnesol-like activities in that they induce a shift to yeast form growth by the fungus [17]. For instance, *P. aeruginosa*–produced 3-oxo-C12-homoserine lactone reaches concentrations in mixed-species biofilms that repress *C. albicans* filamentation [17]. By responding to these signaling molecules, the fungus may disperse from sites where other co-inhabitants such as antifungal-producing bacteria are present, conferring a potential selective advantage. Conversely, in mixed biofilms of *S. gordonii* and *C. albicans* (Figure 1A), a bacterially secreted diffusible signal enhances hyphal development by relieving the effects of farnesol on the fungus [7]. Moreover, cell wall–derived molecules such as bacterial muramyl dipeptides induce *C. albicans* hyphal growth, which may also promote fungal invasion of host tissues and virulence [18]. These findings support the concept that both eukaryotic and prokaryotic microorganisms sense and respond to the diverse diffusible signaling molecules produced in the niches where they coexist. Furthermore, we may find that the chemical warfare between bacteria and fungi leads to increased toxin production and increased host damage and inflammation.

## Chemical Interactions between Fungi and Bacteria

In addition to providing attachment sites for different species, bacterial-fungal communities create environmental conditions that promote or control the growth of other microbes. Actively respiring *C. albicans* reduces oxygen tension levels and provides stimulatory factors for streptococci in the oral environment, while the latter provides nutrients that promote fungal growth [1]. In contrast, commensal bacteria that inhabit the female reproductive tract, such as *Lactobacillus* spp. (Figure 1B), inhibit the growth and virulence of *C. albicans* potentially through secretion of organic acids and production of hydrogen peroxide (H_2_O_2_). Supporting these in vitro findings, it has been shown that 96% of healthy women have H_2_O_2_-generating *Lactobacillus* species as part of their microflora, while these bacterial populations are lower in women suffering from vaginosis [8].

## Bacterial-Fungal Interactions Influence Antibiotic Resistance and Host Response to Infection

Mixed bacterial-fungal infections can correlate with increased frequency or severity of disease. In fact, while *C. albicans* is the fourth leading cause of mortality due to systemic infections [1], [19], the risk of mortality may increase upon bacterial and fungal co-infection. *C. albicans* and *Staphylococcus aureus*, for instance, have synergistic effects where mice inoculated with only *S. aureus* show low mortality, whereas co-inoculation with *C. albicans* leads to mortality increases [1], [20]. It is not yet known how the host immune response is perturbed when bacterial and fungal pathogens are both present, but current research seeks to address this important question. The cooperative effects observed in mixed fungal-bacterial infections in vivo could be due to formation of biofilms, since this form of growth can promote resistance to both host clearance pathways and antimicrobial agents. Harriott and Noverr demonstrated that the human pathogen *S. aureus* forms larger biofilms with increased resistance to vancomycin when it is co-cultured with *C. albicans* [19]. Biofilms of *C. albicans* and oral streptococci are similarly more resistant to antibiotics than their single species counterparts [1], [2]. Matrix polymers produced by both organisms might result in a more viscous matrix that is more effective at restricting the penetration of drugs [2], [19]. Understanding the physiology of bacteria and fungi coexisting within mixed microbial communities will greatly aid our ability to effectively treat opportunistic polymicrobial infections and to modulate the behavior of potentially pathogenic bacteria and fungi in beneficial ways.
